# The *Mycobacterium tuberculosis* β-oxidation genes *echA5* and *fadB3* are dispensable for growth *in vitro* and *in vivo*

**DOI:** 10.1016/j.tube.2011.06.006

**Published:** 2011-11

**Authors:** Kerstin J. Williams, Helena I. Boshoff, Nitya Krishnan, Jacqueline Gonzales, Dirk Schnappinger, Brian D. Robertson

**Affiliations:** a Centre for Molecular Medicine & Infection, Department of Medicine, Flowers Building, Imperial College London, South Kensington, London SW7 2AZ, United Kingdom; b Tuberculosis Research Section, Laboratory of Clinical Infectious Diseases, National Institute for Allergy and Infectious Diseases, National Institutes of Health, Bethesda, MD, United States; c Department of Microbiology and Immunology, Weill Cornell Medical College, New York, NY, United States

**Keywords:** *Mycobacterium tuberculosis*, β-Oxidation, Essential, Fatty acids, *echA5*, *fadB3*

## Abstract

There are several lines of evidence pointing towards the importance of β-oxidation in host survival of *Mycobacterium tuberculosis* including enormous gene redundancy for this process; approximately 100 genes are annotated as β-oxidation genes for the five biochemical reactions that break down fatty acids into acetyl-CoA. Although most of these genes are predicted to be non-essential, two of the genes (*echA5* and *fadB3*) are annotated as essential for growth *in vitro*, and therefore could be considered as putative drug targets. However, here we report the construction of *echA5* and *fadB3* null mutants confirming they are non-essential. No significant difference in growth between the mutant and parent strains was observed in either standard Middlebrook medium or in minimal medium supplemented with various carbon sources. Macrophage survival and mouse infection studies also showed no significant difference between the mutant and parent strains. Therefore, we conclude that these genes are dispensable for growth *in vitro* and *in vivo*.

## 1 Introduction

Tuberculosis (TB) is the leading cause of death from an infectious disease worldwide and, although still primarily a disease of developing countries, increased incidence of drug-resistant strains, increased immigration and the worldwide HIV epidemic has caused a gradual increase in the number of TB cases in developed countries.^1^ More alarming is the rise in the global incidence of multiple drug-resistant strains of *Mycobacterium tuberculosis* (MDR-TB), the causative agent of TB. In some parts of the world, one in four people with tuberculosis becomes ill with a form of the disease that can no longer be treated with standard drugs.^2^ Treatment of such people relies on the use of less potent, more expensive second line agents which require longer treatment times (up to two years or more) and possess unpleasant side effects. There are also reports of totally drug-resistant (TDR) TB strains emerging.^3^ Therefore, there is an urgent need for new and improved TB drugs that are active against drug-resistant strains with novel modes of action. This requires a concerted effort of drug target identification, thorough *in vivo* validation of drug targets and the identification of novel inhibitors.

The success of *M. tuberculosis* as a pathogen lies in the ability of the bacilli to replicate and persist in discrete microenvironments within a mammalian host for long periods. In order to do so, the bacilli must acquire and metabolise nutrients from surrounding host tissue. Identification of genes that are essential for intracellular survival could therefore ultimately lead to the development of new TB drugs that shorten treatment regimens. Growth on fatty acids as sole carbon source requires the β-oxidation pathway and gluconeogenesis via the glyoxylate shunt. There is much evidence to suggest that these fatty acid utilisation pathways play an important role in host survival of mycobacteria such as the extensive lipid degradation gene duplication (∼100 genes annotated as β-oxidation genes for the 5 reactions).^4^ Further evidence includes the observation that fatty acids were shown to be the preferred carbon source for mycobacteria isolated from animal lungs,^5^ deletion of *M*. *tuberculosis* isocitrate lyase 1 and 2 genes (key enzymes of the glyoxylate cycle) caused severe growth inhibition of *M*. *tuberculosis* in mice,^6^ the up-regulation of fatty acid metabolism pathway genes in nutrient starvation models and in macrophage and mice infection studies^7,8^ and the presence of lipid bodies in *M. tuberculosis* isolated from TB patient sputum.^9^

**Figure 1:**
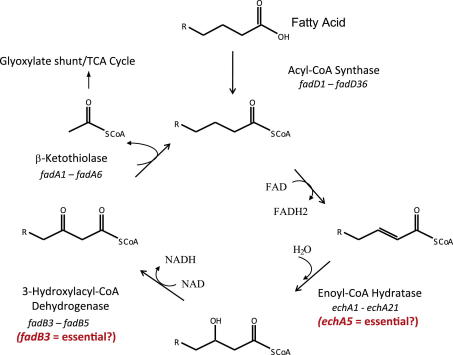
The *M. tuberculosis* β-oxidation genes/pathway. Through successive rounds of oxidation, fatty acids are broken down to produce acetyl-coA which is assimilated, via the glyoxylate shunt, into the TCA cycle. The *echA5* and *fadB3* genes are indicated.

In bacteria, saturated fatty acids are oxidized through β-oxidation to produce acetyl-CoA, which feeds into the tricarboxylic acid (TCA) cycle, and a fatty acid that is two carbons shorter in length which is successively metabolized by this pathway (Figure 1).^10^ The *M. tuberculosis* genome contains over 100 genes annotated as enzymes involved in the five enzymatic reactions of β-oxidation, whereas *E. coli* only has one enzyme set for each of the β-oxidation pathway reactions under either anaerobic or aerobic conditions.^10^ It has been postulated that the gene redundancy observed in *M. tuberculosis* allows the bacilli to adapt and survive by switching their metabolism to utilise the available carbon sources encountered in the various environments *in vivo*. However, this has not been investigated. Although the majority of β-oxidation genes are annotated as non-essential *in vitro*, possibly due to extensive gene redundancy, two have been annotated as essential for *in vitro* growth (Figure 1) based on saturating transposon mutagenesis studies^11,12^ arguing against complete redundancy of these two enzymatic steps.

Enoyl-CoA hydratase [EC 4.2.1.17] catalyses the third step in the β-oxidation pathway, and *echA5* is the only enoyl-CoA hydratase out of 21 encoded in the *M. tuberculosis* genome thought to be essential *in vitro*. The enzyme facilitates the addition of a water molecule to an enoyl-CoA thioester, resulting in the formation of a β-hydroxyacyl-CoA thioester (Figure 1). Studies have shown some of the *M. tuberculosis echA* genes to have altered expression in different environments. For example, in nutrient starvation models *echA8*, *echA11* and *echA12* were shown to be up-regulated whereas *echA10* and *echA21* were down-regulated.^13,14^ In macrophage infection studies *echA19* was up-regulated^7^ whereas *echA7* was up-regulated in mouse infections^15^ suggesting different roles for at least some of the *echA* paralogues. However, to date there are no reports on the role of *echA5* or the altered expression of *echA5* in stress conditions or models of infection in the literature.

The enzyme encoded by *fadB3,* encoding a β-hydroxyacyl-CoA dehydrogenase [EC 1.1.1.35], catalyses the fourth step of the β-oxidation pathway, and is the only hydroxyacyl-CoA dehydrogenase enzyme out of the five encoded in the *M. tuberculosis* genome thought to be essential *in vitro*.^11,12^ Studies have shown the *M. tuberculosis fadB2* and *fadB3* genes to be up-regulated in macrophage infections and *fadB2* up-regulated in mouse infection studies.^7,15^ The *fadB2* gene was also up-regulated in low pH and SDS shock,^16,17^ and in the nutrient starvation model *fadB* and *fadB4* were up-regulated.^13,14^ Furthermore, *fadB5* was found to be deleted (partially or completely) in one or more clinical isolates,^18^ although the significance of this is unclear. This also suggests different roles for the five *fadB* paralogues *in vivo*.

In order to investigate the proposed *in vitro* essentiality of *echA5* and *fadB3* and to explore their roles further, null mutant construction were attempted. Rather unexpectedly, null mutants for both genes in *M. tuberculosis* could be obtained. Therefore, we probed the function of these genes further by *in vitro* growth analysis of the parent and mutant strains on various sole carbon sources. Additionally, the ability of the mutant strains to infect and survive within bone-marrow derived mouse macrophages and to cause infection within the mouse model was investigated.

## 2 Materials and methods

### 2.1 Bacterial strains and growth conditions

*M. tuberculosis* H_37_Rv was grown in Middlebrook 7H9-Tw-Glycerol-OADC liquid broth (supplemented with 0.2% glycerol, 0.05% Tween-80 and 10% OADC) at 37 °C shaken at 150 r.p.m. in an orbital shaker, on Middlebrook 7H11 solid media supplemented with 0.5% glycerol and 10% OADC (Becton Dickinson) or in Sauton’s Minimal Medium supplemented with 0.2% glycerol, 0.0001% ZnSO_4_ and 0.025% Tyloxapol as described previously.^19^ For growth with various carbon sources, Sauton’s minimal medium (without glycerol) was supplemented with the final concentration of carbon source as follows: 0.2% glucose, 10 mM sodium butyrate, 1 mM sodium acetate, 0.25 mM cholesterol, 5 mM sodium propionate, 10 mM sodium pyruvate, 0.1 mM sodium stearate, 0.1 mM isopalmitate, 0.1 mM palmitate, 0.1 mM Dipalmitoyl-phosphatidylcholine (DPC) and 0.1 mM Methyl-arachidonate (MA). All fatty acid sodium salts were prepared by dissolving the powder in water except for palmitate, isopalmitate, stearate, cholesterol, DPC and MA which were dissolved in a 1:1 Tyloxapol:Ethanol mix at 80 °C heat as described previously.^20^ All *E. coli* strains were grown on LB-agar plates or in LB broth. Hygromycin (*Invitrogen*) was added as required at a concentration of 200 μg ml^−1^ for *E. coli* and 50 μg ml^−1^ for mycobacteria. Kanamycin (Sigma) was added at a concentration of 50 μg ml^−1^.

### 2.2 Plasmids and DNA work

Plasmids were generated by using standard cloning procedures. The correct sequence of all cloned PCR fragments was confirmed by DNA sequencing. Genomic DNA was prepared as described previously.^21^ PCR was performed using Qiagen High Fidelity Taq polymerase according to manufacturer’s instructions with the addition of 5% DMSO.

### 2.3 Construction of *echA5* and *fadB3* null mutants

The mycobacteriophage-based method of specialized transduction, which utilizes conditionally replicating shuttle phasmids created from pHAE159 and pYUB854, was used to create the *M. tuberculosis* mutants. Initially, upstream (UP) and downstream (DN) sequences flanking the genes to be mutated were amplified from *M. tuberculosis* genomic DNA by PCR as stated. Primer sequences are listed in Table 1. The flanking regions were designed so as not to disrupt any neighbouring predicted essential genes or to introduce any downstream effects. The resulting knock-out plasmids pDEKO (*echA5*) and pDFKO (*fadB3*) were then used in accordance with the method by Bardarov et al.^22^ to generate the recombinant phage for transduction into *M. tuberculosis*. Putative null mutants were selected on 7H11 agar containing hygromycin (50 mg ml^−1^).

### 2.4 Confirmation of mutant construction

Confirmation of gene deletion was carried out by PCR on gDNA using primers outside the upstream and downstream flanking regions in combination with hygromycin cassette specific primers (Table 1). PCR products would only be obtained with insertion of the hygromycin cassette by recombination onto the chromosome at the correct location. PCR over the disrupted region was also performed. Further confirmation was provided by Western analysis using a custom made EchA5 peptide-specific polyclonal antibody (Eurogentec, Belgium).

### 2.5 Growth analysis of strains

*M. tuberculosis* wild type and mutant strains were grown to mid-log phase (OD 0.8–1) in 7H9 media as previously stated. The cells were then washed twice with PBS, resuspended in PBS and used to inoculate fresh medium (7H9-Tw-Glycerol-OADC or Sauton’s minimal medium plus required carbon source) to a starting OD_600_ of 0.05. Growth was analysed by taking OD_600_ readings. All analyses were performed in triplicate except where stated.

### 2.6 *In vitro* infection of bone-marrow derived murine macrophages

Macrophages were derived from mouse bone-marrow cells by culture for 7 days in RPMI 1640 (Lonza) supplemented with 1 mM sodium pyruvate (Lonza), 2 mM l-glutamine (Lonza), 0.05M 2-mercaptoethanol (Gibco), 10% heat-inactivated foetal bovine serum (Biosera) and 20% L-cell conditioned media. Macrophages were seeded into 24-well tissue culture plates at a concentration of 5 × 10^5^ cells per well and allowed to adhere overnight. *M. tuberculosis* strains were grown to mid-exponential phase, pelleted by centrifugation, washed twice with PBS-Tween, once with PBS and resuspended in RPMI to a concentration of 5 × 10^5^ cfu ml^−1^. The bacteria were added to the monolayers at a multiplicity of infection (m.o.i.) of 1 for 4 h at 37 °C in triplicate wells. The medium was removed and replaced with RPMI medium containing 200 μg ml^−1^ amikacin and incubated for 45 min at 37 °C. The monolayers were then washed three times with RPMI to remove free bacteria and then cultured in RPMI at 37 °C with 5% CO_2_. Intracellular survival and growth was assessed by lysis of the monolayers by the addition of water followed by a 30 min incubation at room temperature and enumeration of bacteria by serial dilution in PBS-Tween plating onto Middlebrook 7H11 solid medium supplemented with 0.5% glycerol and 10% OADC. Colonies were counted after 3–4 weeks incubation at 37 °C and the average cfu/well determined.

### 2.7 Mouse infections

Eight-week-old C57Bl/6 mice were infected with 100–140 colony forming units of *M. tuberculosis* H_37_Rv parental strain or the 2 gene knock-out strains by aerosol using a BioAerosol nebulizing generator (CH Technologies Inc., Westwood, NJ) for 10 min. Groups of 5 mice for each strain were euthanized on days 1, 14, 28 and 56 and lungs and spleens homogenized in Middlebrook 7H9-Tw-Glycerol-OADC liquid broth. Appropriate dilutions were plated on Middlebrook 7H11 solid medium supplemented with 0.5% glycerol and 10% OADC in duplicate and incubated at 37 °C for colony enumeration.

## 3 Results

### 3.1 Construction and confirmation of *echA5* and *fadB3 M. tuberculosis* null mutants

**Figure 2:**
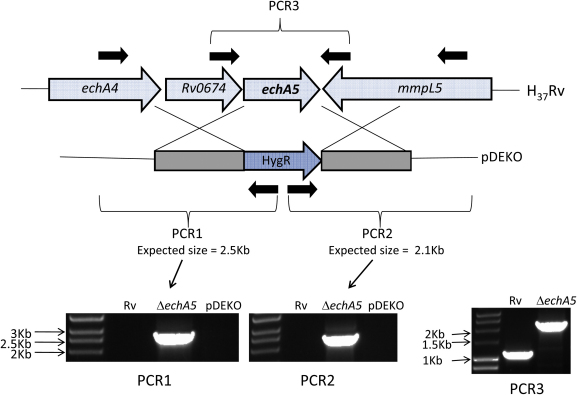
Confirmation of *echA5* null mutant genotype by PCR. PCR products of the expected size were obtained for the mutants with pYB_HygOut1 and *echA4*_F oligos (PCR1), and using pYB_Out2 and mmpL5_F oligos (PCR2); no products were obtained with wild type or knock-out plasmid DNA (PCRs 1 & 2). PCR over the *echA5* gene using *echA5*_ko_screen oligos further confirms the correct insertion of the hygromycin cassette (PCR3).

**Figure 3:**
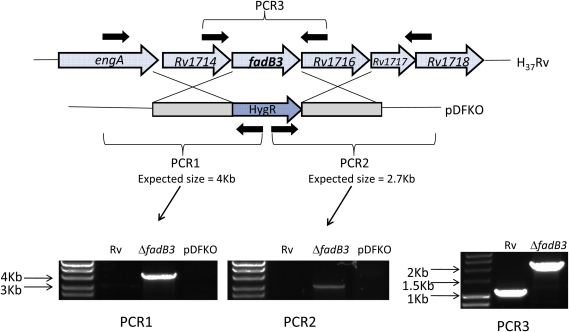
Confirmation of *fadB3* null mutant genotype by PCR. PCR products of the expected size were obtained for the mutants with pYB_HygOut1 and *engA_*F oligos (PCR1), and using pYB_Out2 and Rv1718_R oligos (PCR2); no products were obtained with wild type or knock-out plasmid DNA (PCRs 1 & 2). PCR over the *fadB3* gene using *fadB3*_ko_screen oligos further confirms the correct insertion of the hygromycin cassette (PCR3).

The method of Bardarov et al.^22^ was used to attempt mutant construction by allelic exchange in *M. tuberculosis*. Unexpectedly, mutant strains for both genes could be obtained. Allelic exchange was confirmed by PCR using oligonucleotides specific to the hygromycin cassette and to the genomic region outside of the target-gene flanking regions used to construct the mutants. PCR products would only be obtained if the hygromycin cassette had inserted into the correct location on the chromosome. PCR products of the expected size were obtained for the null mutants; no products were obtained for the wild type parent strain or the plasmid used to construct the mutants (Figures 2 and 3). PCR over the region of disruption further confirmed mutant construction by showing an increase in size corresponding to insertion of the hygromycin cassette at this location (Figures 2 and 3). Additional confirmation that the *echA5* gene has been disrupted was provided by Western blot showing the absence of the corresponding 27.5 kDa protein in the mutant strain compared to the wild type (Supplementary Data Figure S1). Antibody was not available for FadB3 Western analysis.

### 3.2 Initial *in vitro* growth analysis of parent and mutant strains in standard growth medium

**Figure 4:**
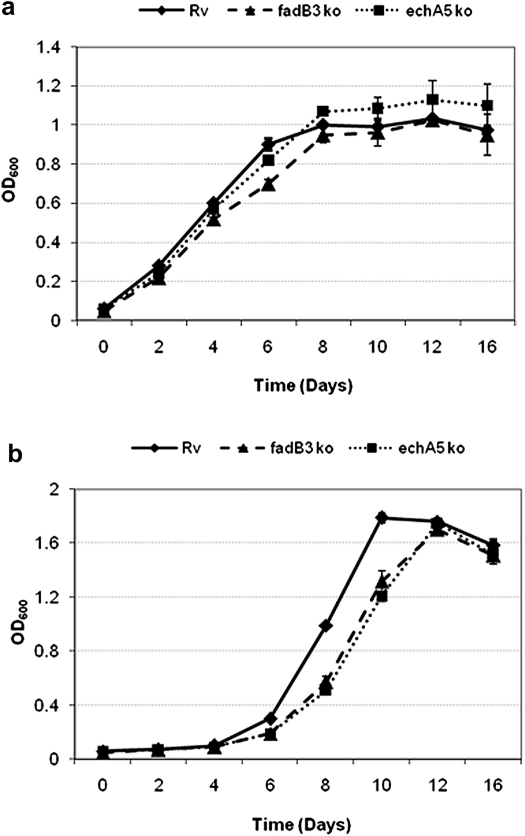
Growth kinetics of *M. tuberculosis* (H37Rv), *echA5* mutant and *fadB3* mutant in (a) Middlebrook 7H9-Tw-Glycerol-OADC medium and (b) Sauton’s minimal medium plus 0.2% glycerol. Results shown are the average of three independent experiments. Error bars represent SD.

The β-oxidation null mutant strains showed no growth defect compared to wild type when grown in 7H9 medium (Figure 4a). However, this medium contains many carbon sources and it was therefore investigated whether any potential growth defect of the mutant was being masked by the presence of other fatty acids and carbon sources in the medium. For example, the Middlebrook 7H9-Tw-Glycerol-OADC medium used for propagation of these strains contains glycolytic carbon sources in the form of glycerol and glucose as well as the β-oxidation substrate oleic acid, including the oleic acid released during hydrolysis of Tween-80. Therefore growth analysis of the mutants in Sauton’s minimal medium where the surfactant Tween-80 was replaced with the non-hydrolyzable surfactant Tyloxapol with glycerol as the sole carbon source was performed (Figure 4b). Sauton’s minimal medium with no exogenous carbon source was included as a negative control in all growth studies. The mutants grew at a slightly slower rate on glycerol compared to wild type, but could still utilise glycerol as a sole carbon source and therefore in terms of the essentiality of these genes for growth using glycerol as a sole carbon source this was not deemed significant.

### 3.3 Further *in vitro* growth analysis of parent and mutant strains in various carbon sources

In order to investigate the role of these genes further, growth in Sauton’s minimal medium supplemented with a variety of fatty acids was tested. Growth was observed for all strains in glucose, propionate, butyrate, stearate, cholesterol, acetate, palmitate, isopalmitate, Dipalmitoyl-phosphatidylcholine (DPC), Methyl-arachidonate (MA) and pyruvate; although the growth rate varied between each carbon source, there was no significant difference in growth rate between wild type and mutant strains (Supplementary Data Figure S2). As was observed with glycerol as the sole carbon source, the mutants grew at a slower rate on glucose compared to wild type, however in terms of the essentiality of the genes for growth on glucose this was not deemed significant.

### 3.4 Survival of parent and mutant strains *in vivo*

**Figure 5:**
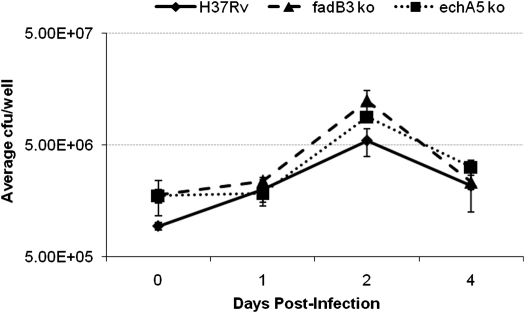
The intracellular growth of the parent and mutant strains within mouse bone-marrow derived macrophages. Results shown are the average of triplicate wells and are representative of two independent experiments. Error bars represent SD.

**Figure 6:**
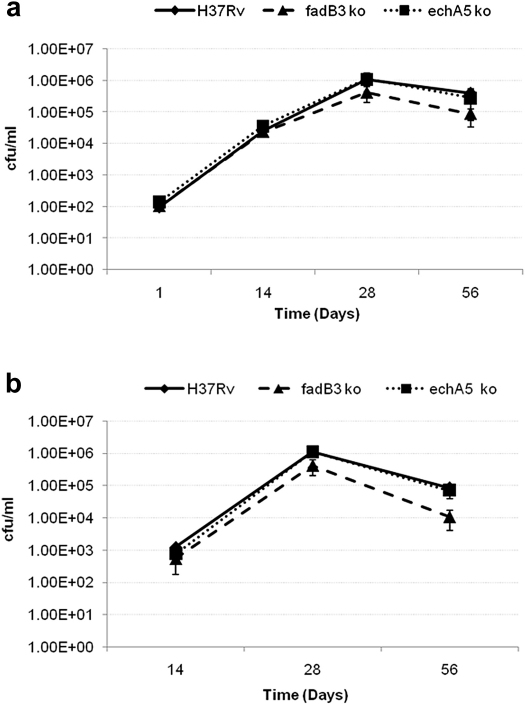
*In vivo* growth and survival of the parent and mutant strains. Growth was monitored in the lungs (a) or the spleen (b) over 8 weeks post-infection. Results shown are the median cfu/ml from groups of five mice. Error bars represent SD.

To test the importance of *echA5* and *fadB3* during intracellular growth in host cells, we infected mouse bone-marrow-derived macrophages with wild type, *echA5* ko mutant and *fadB3* ko mutant strains at a multiplicity of infection (m.o.i.) of 1. All three bacterial strains showed intracellular growth over the first 2 days post-infection and by day 4 post-infection a slight reduction in intracellular growth had occurred, however no difference in growth or survival between the parent and mutant strains was observed (Figure 5). Similarly, there was no defect in growth or survival of these strains in the lung or the spleens of infected mice up to 8 weeks post-infection (Figure 6).

## 4 Discussion

Metabolic adaption to the host niche is critical for the survival and pathogenicity of *M. tuberculosis.* Although bacteria can utilise a variety of carbon sources for growth *in vitro*, fatty acids are thought to provide the major source of carbon for growth during intracellular infection.^4,7,8,10^ The fatty acid(s) available as carbon sources will vary depending on the host environment the bacilli reside in and therefore it has been proposed that the enormous gene redundancy observed in the *M. tuberculosis* genome for the enzymes involved in this process is to allow the bacilli to adapt and metabolise the available carbon source either aerobically or anaerobically. This hypothesis is further supported by the observation that the non-pathogenic enteric bacteria *E. coli* genome only encodes one set of genes for either aerobic or anaerobic β-oxidation of fatty acids.^5,7,8^

The majority of *M. tuberculosis* genes in the β-oxidation pathway are annotated as non-essential *in vitro*.^11^ However, the exact role of the annotated β-oxidation enzymes in fatty acid utilisation *in vitro* and *in vivo* is not known. There is currently very limited biochemical investigation into the precise role of most of the enzymes in the β-oxidation pathway and therefore some genome annotation mistakes cannot be completely ruled out. In this study, we investigated the role of two β-oxidation genes thought to be essential for growth *in vitro* because transposon mutants for these two genes have not been reported in two independent mutagenesis screens^11,12^ and are not available via target (http://webhost.nts.jhu.edu/target/). However, null mutants could be obtained and therefore we confirmed that these genes are in fact non-essential *in vitro*. There are alternative explanations for the lack of genetic disruption observed in a transposon screen other than essentiality. For example, the transposon library may not be completely saturated, the transposon may not be able to insert itself at a particular genomic region due to secondary structure and the genetic location of the gene may be significant (if a gene is situated in an operon upstream of essential genes then this gene will not be disrupted although may not itself be essential). *fadB3* is in an operon with putative essential genes upstream but only non-essential genes downstream. *echA5* is the last gene of an operon with a putative essential gene upstream, so it is unclear as to why a transposon mutant was not obtained for these genes. This highlights the need to confirm essentiality predictions by targeted mutagenesis.

In order to explore the possibility that the paralogues play a role in utilising different carbon sources, the growth of the mutant strains in a variety of different fatty acids as sole carbon sources was monitored. The depletion of glycerol and glucose in a nutrient starvation model induced 13 β-oxidation genes^14^ and it is therefore thought that the bacilli switch to the β-oxidation of fatty acids to drive the supply of central metabolites via the glyoxylate shunt and gluconeogenesis. Therefore, a range of carbon chain length fatty acids was chosen for testing in this study and cholesterol, thought to be important in bacilli host infection and persistence,^20,23^ was also studied. However, the mutants grew on all of the carbon sources tested, suggesting that these genes are not solely responsible for the metabolism of these carbon sources. Although gene redundancy may be masking any mutant phenotype, we have not fully recapitulated all the potential substrates the bacillus may encounter *in vivo* in this study and a unique role of the *echA5* and *fadB3* genes in the metabolism of other fatty acids cannot be completely ruled out. Furthermore, null mutants *in vitro* undergo many generations of growth during which compensatory mutations and/or gene expression changes may occur to overcome the loss of the gene in that environment.

*M. tuberculosis* displays a significant transcriptional response with more than 600 genes having altered expression levels upon phagocytosis by a macrophage.^7^ In the phagosome, the bacilli are presumably exposed to a limited availability of nutrients, yet they can replicate and persist within the phagosome. Therefore, we tested the ability of the mutant strains to grow and survive within macrophages. Once again no difference was observed with growth occurring for all strains up to 2 days post-infection with a slight drop in survival by day 4. Although the lack of phenotype in the mutant strains is perhaps not unsurprising given the enormous gene redundancy, *fadB3* has previously been shown to be up-regulated in macrophage infections,^7^ however, we show in this study that it is not required for intracellular survival. It is thought that some central metabolism genes may be up-regulated as a general stress response^24^ and this and/or the possible gene redundancy (*fadB2* is also up-regulated in macrophage infection^7^) may account for lack of intracellular phenotype with the *fadB3* mutant. There has been no reported altered *echA5* gene expression in the macrophage model and we confirm it is not individually required for macrophage survival. Similarly for the mouse infection studies, no difference was observed between the parent and mutant strains for infection of the lungs or spleen up to 8 weeks post-infection. In a genetic screen for genes required for growth *in vivo*, neither *echA5* nor *fadB3* were predicted to be required for survival in the mouse model.^25^ However, these mutants may not have been included in the pool of mutants investigated as no transposon mutant was obtained previously leading to the assignment of these genes as essential for *in vitro* growth.^11^ However, eight fatty acid degradation genes were predicted to be required for *in vivo* infection,^25^ further supporting the important role of lipid degradation in the survival of *M. tuberculosis* in the host. However, we observed that *echA5* and *fadB3* are not individually required for survival *in vivo.*

In summary, we show the β-oxidation genes *echA5* and *fadB3* are non-essential for growth *in vitro* or *in vivo* and based on this study we would not consider these genes as viable drug targets. There is an urgent need for new TB treatment shortening drugs with novel modes of action. Very few drug targets make it through to pre-clinical studies as compounds active against the target *in vitro* often have no activity in animal models. Therefore, druggable gene products that are required for *in vivo* survival need to be identified. However, this is not straightforward, since genes essential for *in vitro* growth cannot be studied by conventional knock-out genetics, but instead require more complex conditional expression systems for study. Although initially the β-oxidation pathway genes *echA5* and *fadB3* looked like viable drug targets as they were predicted to be essential *in vitro* and the pathway thought to be important *in vivo*, they turned out not to be attractive drug targets. Although the utilisation of fatty acids via the β-oxidation pathway is still thought to be important for *in vivo* survival of *M. tuberculosis*, the enormous gene redundancy makes biochemical investigation of this pathway difficult. Therefore, the generation of double or multiple gene knock-out strains may be required for further investigations into this pathway’s role in the metabolism and intracellular survival of *M. tuberculosis.*

## Figures and Tables

**Table 1:** Primer sequences used in this study (restriction enzyme sites are underlined).

Name	Sequence	Purpose
*echA5*_KO_Up_F	5′-GATCATCTTAAGTGTTCGACGGCGCCGCTC	Directional cloning of *echA5* upstream region into pYUB854
*echA5*_KO-Up_R	5′-GATCTCTAGACGACCTTTGCGCTCCACACG	“
*echA5*_KO_Dn_F	5′-GATCATAAGCTTAGACGGTTCGCCGCGGGTG	Directional cloning of *echA5* downstream region into pYUB854
*echA5*_KO_Dn_R	5′-GATCACTAGTCGGTTCATCATCAGCCAC	“
*fadB3*_KO_Up_F	5′-GATCATCTTAAGGGACAGCGAGGCCGGTTC	Directional cloning of *fadB3* upstream region into pYUB854
*fadB3*_KO-Up_R	5′-GATCTCTAGACGAGGTCAGCATGCGGTG	“
*fadB3*_KO_Dn_F	5′-GATCATAAGCTTCAAGCCAACGAAAAAGGAAG	Directional cloning of *fadB3* downstream region into pYUB854
*fadB3*_KO_Dn_R	5′-GATCACTAGTGAGCACGACGTAGACGGTC	“
pYB_HygOut1	5′-TTCGAGGTGTTCGAGGAGAC	Confirmation of *fadB3* knock-out – upstream region
*engA*_F	5′-GATTGGCAACTAGACGATTCG	“
pYB_HygOut2	5′-GCATGCAAGCTCAGGATGTC	Confirmation of *fadB3* knock-out – downstream region
Rv1718_R	5′-GTATCGTGCGCGATACCAG	“
pYB_HygOut1	5′-TTCGAGGTGTTCGAGGAGAC	Confirmation of *echA5* knock-out – upstream region
*echA4*_F	5′-CAATTGATCATGGTCAAGCTC	“
pYB_HygOut2	5′-GCATGCAAGCTCAGGATGTC	Confirmation of *echA5* knock-out – downstream region
*mmpL5*_F	5′-GGATCATTCTGGGGATGAACG	“
*echA5_ko_screen_F*	5′-AAAGCGTGTCGCCCTGTTCG	PCR over *echA5* gene
*echA5_ko_screen_R*	5′-ACCTGCTCACCGATCCGATG	“
*fadB3_ko_screen_F*	5′-CGATCTGCACGTCCTCGAAG	PCR over *fadB3* gene
*fadB3_ko_screen_R*	5′-GATCTATCTGCTCAGCGACG	“

## Appendix Supplementary data

Figure S1. Western blot confirming loss of the 27.5 kDa EchA5 protein in the *echA5* null mutant. M = Pre stained ladder; 20 μg total protein (determined by BCA Assay) was loaded in each lane.

Figure S2. Growth kinetics of *M. tuberculosis* H_37_Rv *echA5* mutant and *fadB3* mutant grown with a sole carbon source as stated (*n* = 3 except for ∗*n* = 2). Wild type (continuous line), *fadB3* null mutant (dashed line) and *echA5* null mutant (dotted line). Error bars represent SD.
