# Supplementary figures and images for: The Mycobacterium tuberculosis β-oxidation genes echA5 and fadB3 are dispensable for growth in vitro and in vivo
Source: Tuberculosis (Edinb). 2011 Nov;91(6-3):549–55. doi: 10.1016/j.tube.2011.06.006 (PMC3220763; doi:10.1016/j.tube.2011.06.006)

## Slide 1
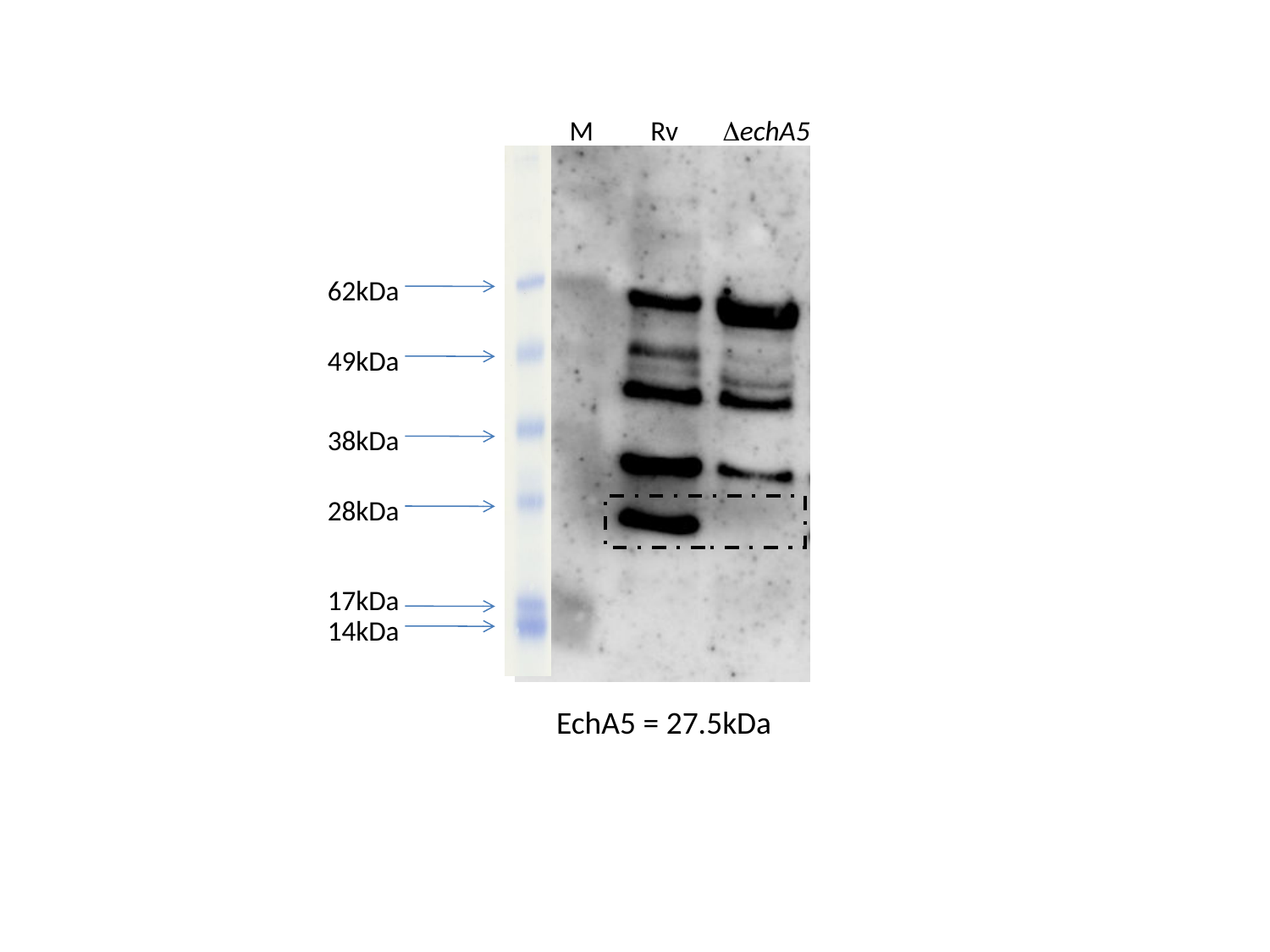

M Rv ΔechA5
62kDa
49kDa
38kDa
28kDa
17kDa
14kDa
 EchA5 = 27.5kDa

Supplement: Supplementary file 1 — Figure S1. Western blot confirming loss of the 27.5 kDa EchA5 protein in the echA5 null mutant. M = Pre stained ladder; 20 μg total protein (determined by BCA Assay) was loaded in each lane. [file mmc1.ppt]

## Slide 1
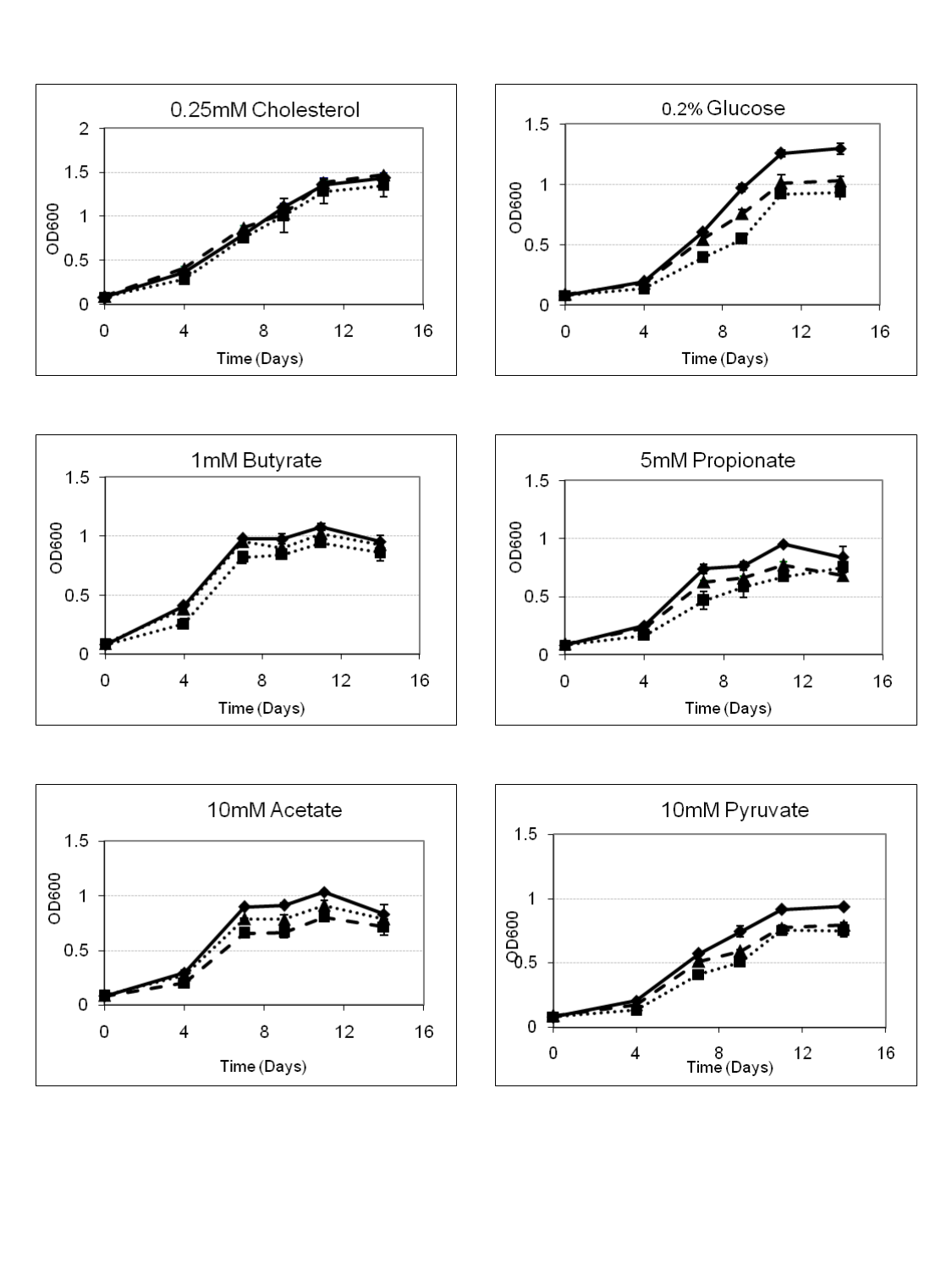

## Slide 2
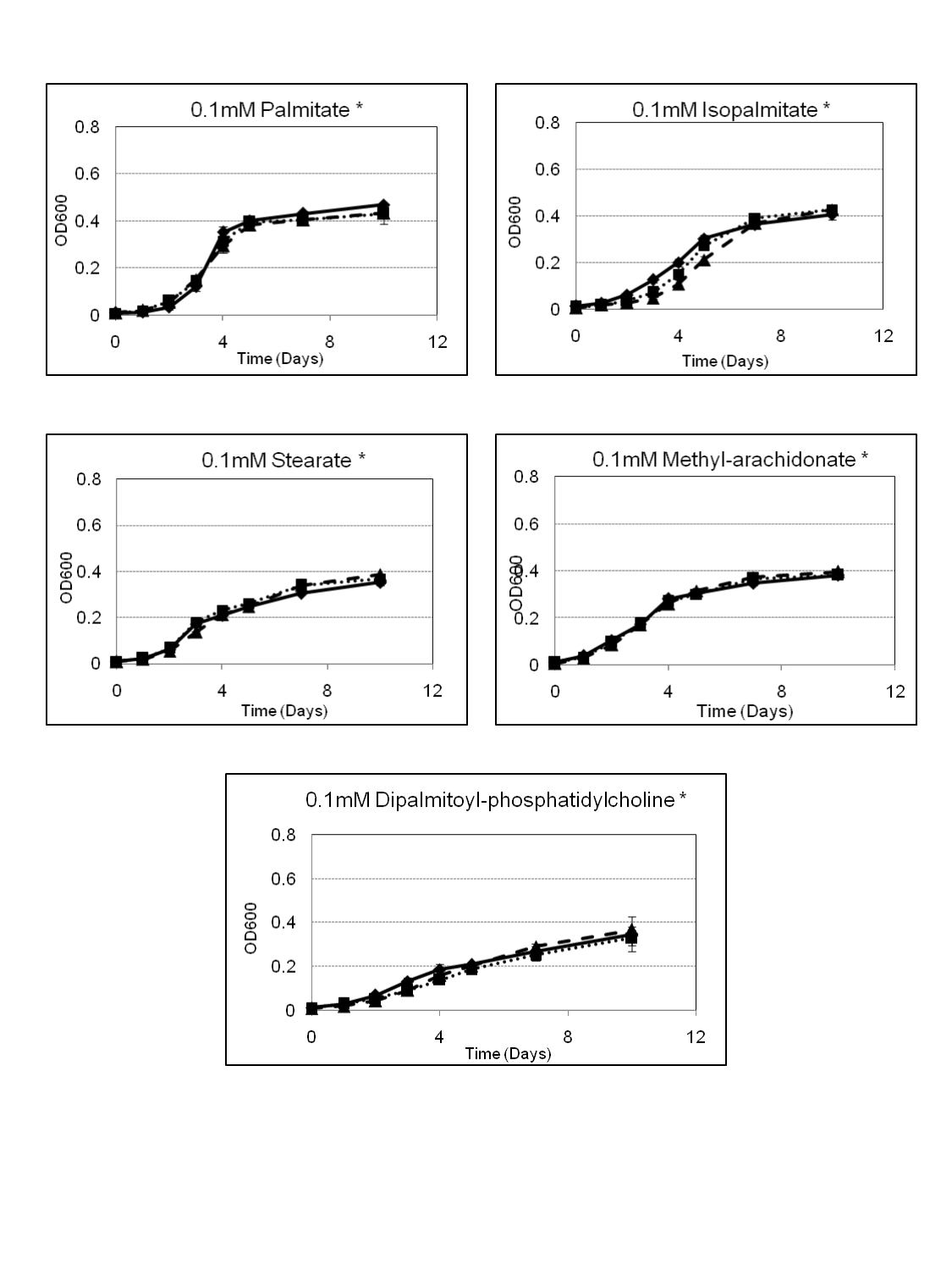

Supplement: Supplementary file 2 — Figure S2. Growth kinetics of M. tuberculosis H37Rv echA5 mutant and fadB3 mutant grown with a sole carbon source as stated (n = 3 except for ∗n = 2). Wild type (continuous line), fadB3 null mutant (dashed line) and echA5 null mutant (dotted line). Error bars represent SD. [file mmc2.ppt]
